# Ultrasound-Guided Peripheral Intravenous Access Training: A Prospective Observational Study of Emergency Nurses

**DOI:** 10.7759/cureus.66705

**Published:** 2024-08-12

**Authors:** Hillary McKinley, Weeden Bauman, Erik Christensen, Peter Croft, David Mackenzie, Samantha Fillebrown, Christina Wilson

**Affiliations:** 1 Emergency Medicine, Maine Medical Center, Portland, USA

**Keywords:** pocus, education, piv, nursing, ultrasound

## Abstract

Background

Ultrasound-guided peripheral intravenous (USGPIV) placement is more successful, comfortable, and longer lasting than traditional landmark-based IVs. While many hospitals have protocols for becoming credentialed in this skill, there is little information available about the USGPIV success rate during and after training.

Objectives

This pilot study aimed to quantify USGPIV attempts by emergency nurses undergoing USGPIV training and to determine if 10 successful USGPIVs predicted success in the next USGPIV. The secondary aims were to determine failure points for unsuccessful USGPIVs and the time to complete training.

Methods

Sixteen emergency nurses with no prior USGPIV experience were enrolled. Participants completed a one-hour didactic session and one hour of hands-on training with vein simulators. Participants had direct observation of all USGPIV attempts up until 10 successful cannulations. Each attempt was recorded along with reasons for USGPIV attempt failure and the time required to become credentialed.

Results

Participants attempted 200 USGPIVs with a success rate of 80% (160/200). Fourteen participants completed 10 successful USGPIVs, requiring a median of 11 attempts (IQR 10-13) and a success rate of 92.9% (13/14) on subsequent USGPIV attempts. Participants required a median observation time of 4.13 hours (IQR: 3.94-4.75). The most common point of failure was using dynamic ultrasound guidance to maintain needle tip visualization.

Conclusions

USGPIVs are a valuable skill that requires time and practice. Emergency nurses with no prior USGPIV experience can achieve the requirements for hospital credentialing and success on subsequent USGPIV insertion by completing 10 successful USGPIVs after a two-hour training session and four hours of direct observation.

## Introduction

More than half of all patients seen in the ED will require the placement of a peripheral intravenous (PIV) catheter [1]. Difficult IV access (DIVA) is common and can lead to delays in diagnosis, treatment, and disposition [2]. Ultrasound can be used to identify target veins and guide needle insertion. Ultrasound-guided PIV (USGPIV) has been shown to be more successful than PIV placed using the traditional landmark technique and can reduce the number of skin punctures, decrease the time needed to achieve access, and improve patient satisfaction [3]. USGPIV insertion can be successfully performed by ED nurses after receiving training [4-8].

Numerous studies have described the USGPIV success rate after completion of a training and credentialing process [4-6,9,10]. There is less research available about the USGPIV success rate during training and the number of attempts and trainee time required to achieve the required number of successful cannulations for ultimate credentialing in the skill [7,8,11-13].

This study primarily aimed to quantify the number of USGPIV attempts made by ED nurses during their training before achieving 10 successful cannulations and to determine the immediate success rate of USGPIV insertion after completion of those 10 successful cannulations. The secondary aims were to determine the failure points for unsuccessful USPIV attempts and to identify the time needed to complete the training and credentialing process.

## Materials and methods

This prospective observational study was conducted at Maine Medical Center, Portland, Maine, USA, a single Level I trauma center with an annual volume of approximately 70,000 patients. In April 2023, in collaboration with the hospital vascular access team, our ED developed a training course for USGPIV training. Full-time ED nurses with more than two years of clinical experience were offered optional training in USGPIV placement. Volunteers completed one hour of USGPIV didactics and one hour of hands-on training with vein simulators taught by two ED attending physicians with fellowship training in point-of-care ultrasound. The didactic session was delivered by a live instructor and focused on vein identification with ultrasound; tips for good vein selection, including depth, location, and orientation; a detailed description of ultrasound-guided technique for IV insertion with a focus on visualization of the needle tip using dynamic short-axis out-of-plane technique; procedure ergonomics; and troubleshooting and machine disinfection. During the hands-on simulation session, the instructor reviewed the basic use of the ultrasound machine. Participants practiced vein identification on a live model and then had unlimited attempts to cannulate a homemade tofu vein simulator.

In order to be credentialed in USGPIV at our institution, nurses must place 10 successful USGPIVs under supervision, not including vascular phantom attempts. To achieve this, each course participant scheduled one-on-one vascular access shifts in the ED with an instructor. No other nursing duties or responsibilities were required; the sole focus was on USGPIV training. Instructors were ED attending physicians with fellowship training in point-of-care ultrasound and experience with USGPIV placement. During these shifts, any patient who had been triaged and required an IV had the trainees attempt USGPIV placement. Eligible patients included any patients identified as having DIVA and those that were not identified as having DIVA. DIVA was defined as patients who had previously required USGPIV, had multiple failed landmark IV attempts, were on dialysis via arteriovenous fistula, or had a history of using IV drugs. Instructors provided verbal guidance and feedback during each attempt but did not touch the ultrasound probe or IV catheter. An attempt was considered successful if the operator used ultrasound to guide the IV catheter into the vein lumen, resulting in an IV that was able to draw back blood and be flushed without tissue extravasation. If a USGPIV was unable to draw back blood but had saline flushed without tissue extravasation, the USGPIV was considered a success. After two unsuccessful attempts, trainee USGPIV attempts were aborted, and the patient either had a landmark-guided IV catheter placed by the nurse or a USGPIV placed by the physician observer.

Eligible study participants were ED nurses who had no prior experience in USGPIV placement and had completed the two-hour didactic and hands-on simulation training. Eligible participants were approached at the completion of the training course and prior to attempting USGPIV placement on live patients. During supervised USGPIV sessions, study investigators documented information on each USGPIV attempt, with an attempt defined as a skin puncture. The collected data included USGPIV success or failure and whether the patient receiving the IV had DIVA. A checklist of key steps to the placement of a USGPIV was created based on the credentialing checklist provided by the hospital vascular access team and expert opinion from ultrasound fellowship-trained faculty (Table 1). Success or failure for each step was recorded for each attempt. The total time spent by the observer was documented for each observation session. This total time included time spent identifying eligible patients, each USGPIV attempt from preparation through dressing and cleaning, and time spent giving additional feedback. After the completion of 10 successful USGPIVs, each participant was observed for one additional attempt. This attempt could be on any patient, not just those categorized as DIVA. Observers did not provide verbal guidance or feedback during this subsequent attempt.

**Table 1:** Checklist for USGPIV placement with potential points of failure USGPIV, ultrasound-guided peripheral intravenous

Checklist for USGPIV	Number of times this step is identified as the cause of USGPIV failure
Linear probe used	0
Tourniquet placed	0
Vessel is identified as a vein	0
Vein depth appropriate	0
Vein size appropriate	0
Needle placed close to the probe for skin entry	2
Needle tip identified in the soft tissue after entry	10
Dynamic ultrasound guidance is used to maintain visualization of the needle tip	15
After vein puncture, dynamic ultrasound guidance used to further insert the needle	8
Catheter threaded into the vein	4
Skin cleaned and IV secured	0

## Results

**Figure 1:**
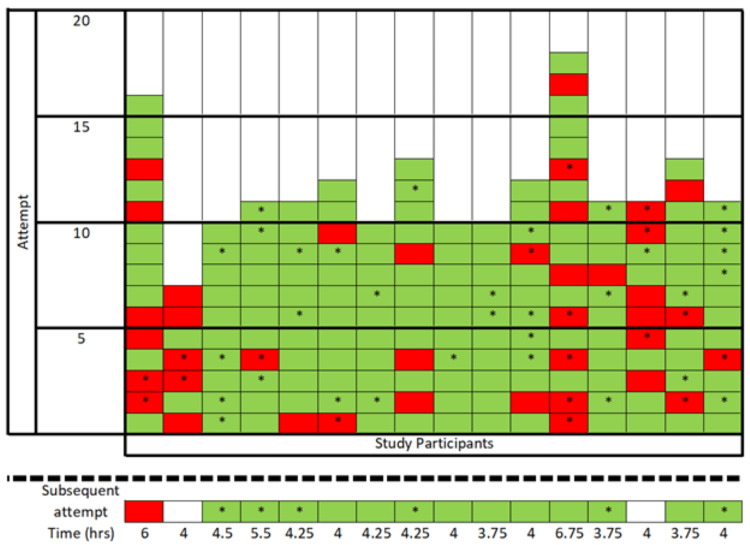
Individual performance of each USGPIV attempt during direct observation and subsequent USGPIV attempt after 10 successful insertions The total observer time is listed for each subject. * = DIVA; green = successful USGPIV; red = unsuccessful USGPIV; white = no attempt DIVA, difficult intravenous access; USGPIV, ultrasound-guided peripheral intravenous

A total of 23 nurses completed the two-hour prerequisite training session. Three nurses were excluded from the study as they had prior experience with USGPIVs. Four nurses completed the two-hour prerequisite training but did not initiate the credentialing process and were also excluded from the study. The remaining 16 eligible nurses all consented to participation. The 16 eligible nurses attempted a total of 200 USGPIVs during the training phase and were directly observed (Figure 1). The overall USGPIV success rate during training was 80% (160/200). Of all USGPIV attempts, 28% (56/200) were considered DIVA, and USGPIVs attempted in this subgroup had a success rate of 67.9% (38/56). The most common causes of unsuccessful attempts were failure to identify the needle tip in the soft tissue after entry and failure to use dynamic ultrasound guidance to maintain visualization of the needle tip (Table 1).

Fourteen out of 16 participants achieved hospital credentialing by performing 10 successful USGPIVs, and this required a median of 11 attempts (IQR 10-13) (Figure 1). The two participants who did not achieve 10 successful USGPIVs did not respond to email requests to schedule additional training time with the instructors. The highest number of USGPIV attempts required to achieve 10 successful placements was 18, and four participants were successful on all 10 of their attempts. Among the 14 participants who achieved 10 successful USGPIVs, the subsequent USGPIV was observed with an overall success rate of 92.9% (13/14) with a success rate of 100% (6/6) in patients classified as DIVA.

Participants required a median observation time of 4.13 hours (IQR: 3.94-4.75) to achieve 10 successful USGPIVs with a range of 3.75 hours to 6.75 hours. The time is calculated from the time the nurse signed into the observation shift until the clean-up of the last USGPIV. This was thought to best capture all aspects of the USGPIV training session, including finding patients, setting up for the procedure, completing the procedure, and discussing feedback with the trainer, to make this a realistic metric of training time. Given the secondary aim of the study, the authors sought to quantify a realistic estimate of trainee and instructor time required for these directed observation sessions. With the addition of the prerequisite two-hour didactic and simulation training, the median time for completion of USGPIV training was 6.13 hours.

## Discussion

The nurses in this study achieved a USGPIV success rate of 80% during the credentialing phase, with an immediate post-training success rate of 92.9%. Previous studies have had highly variable post-training success rates, ranging from 64.7% to 97.5%, with our success rate falling on the higher end [4-6,9,14-16]. Trainees in our study had 100% success on their first post-training USGPIV attempt on patients with DIVA, although the number of participants in this study was small. Our training did not specifically focus on placement in patients with DIVA and allowed the trainees to practice on all patients. The reasoning behind this was twofold: one, allowing attempts on all patients allowed the training to proceed more quickly as trainees did not have to wait for a patient with DIVA to be available, and two, we reasoned that placing a USGIV in a patient with more vein availability and easier targets may be an easier situation in which to learn and develop skills than in a higher stress scenario in which there are more challenging targets. The skills learned on patients without DIVA translated very well to patients with DIVA, suggesting that the learning phase does not need to specifically focus on patients with DIVA.

This study was not designed to determine the most appropriate number of successful USGPIVs that should be required for credentialing in this skill, but it did offer interesting insight. We required 10 successful placements based on our hospital’s credentialing policy and found that our trainees were able to achieve 10 successful cannulations after a median of 11 attempts. Prior studies have used a requirement of five to 10 successful attempts [4,6,9,10,12,15] but some have used as few as one [5] or as many as 40 [12,14] to complete training requirements. A multicenter study found that a mean number of 34 procedures was required to achieve competency in their trainees [12]. Other studies have shown that the success rate for new learners increases steadily with an increasing number of USGPIV attempts, with their trainees achieving high rates of success after 15-30 attempts [4,7]. In a study by Ault et al., trainees required an average of 25 attempts to achieve 10 successful USGPIV placements [11]. In our study, four of the participants had a 100% success rate throughout the training, while others struggled more with the skill and had failed attempts late into the training. This argues that an arbitrary cutoff number is unlikely to be applicable to all learners. Some learners may be able to achieve competence faster than others, and perhaps the number of required successful USGPIVs should allow flexibility based on individual progress and skill demonstration.

The most common causes of unsuccessful USGPIV attempts in our study were failure to use dynamic ultrasound guidance to maintain visualization of the needle tip, failure to identify the needle tip in the soft tissue after entry, and failure to use dynamic ultrasound guidance to further insert the needle into the vein. Failure points have not been previously described. Based on our data, more time should be spent during the didactics and initial simulation training session focusing on visualization of the needle tip and dynamic guidance. Additionally, future phantom models could be created to more realistically simulate veins with an oblique course or with a bifurcation to challenge needle guidance skills.

USGPIV training programs are not standardized, although most share similarities. A systematic review of teaching methods identified that 97.1% of published training programs are multimodal, including didactics, simulation, and proctored IV insertion in live patients [14]. Didactics range from 30 minutes to three hours, and hands-on simulation training is one to two hours on average, with some protocols involving up to eight hours [16]. Very few studies clarify the structure or timeline for the proctored IV insertion portion of the training. One study described the use of one-on-one, four- to eight-hour sessions for proctored USGPIV insertion [16]. We were able to schedule four-hour shifts in which the trainees had no other responsibilities. This allowed the trainees and instructors to focus on spaced repetition and skill acquisition. We think that the one-on-one time with an instructor contributed to our high USGPIV success rate by allowing for intensive and individualized verbal feedback during and after each insertion. This model required time commitment from both trainees and instructors outside of their normal working hours but also allowed trainees to complete their credentialing process quickly. Although effective in a small pilot group, this training model has been difficult to scale to a large group of learners given the one-on-one instructor time required.

Limitations

There are several limitations to this observational study. This was a single-center pilot study with a very small sample size, making the results difficult to generalize. Study participants volunteered to complete the training session and to enroll in the study, which likely introduced bias as nurses who chose to dedicate time to additional training may have been more committed to learning than if the training was required. Additionally, participants were likely to have variable previous experience with landmark-guided IVs. Due to scheduling restraints, including the availability of participating nurses and the study investigators, we were only able to observe one additional attempt after the completion of 10 successful IVs. Further studies observing numerous subsequent attempts would be helpful. There was a variable number of DIVA and non-DIVA patients. Ideally, this could be standardized to create the same training experience for all participants. Due to time constraints and motivation to help nurses complete training in an efficient manner, we were unable to require a specific number of DIVA attempts.

## Conclusions

This observational pilot study showed that ED nurses with no prior USGPIV experience can quickly achieve 10 successful USGPIVs after a median of 11 attempts and have a success rate of 92.9% on their subsequent attempts. This study did not quantify the ideal number of USGPIV attempts to achieve competency in this skill but did show that 10 is a reasonable number for a good next-attempt success rate and can be achieved through two hours of didactic training and four hours of direct observation with an experienced instructor.
